# Developing a Follow-Up Strategy for Patients with PSA Ranging from 4 to 10 ng/ml via a New Model to Reduce Unnecessary Prostate Biopsies

**DOI:** 10.1371/journal.pone.0106933

**Published:** 2014-09-30

**Authors:** Ruizhe Zhao, Yuan Huang, Gong Cheng, Jinliang Liu, Pengfei Shao, Chao Qin, Lixin Hua, Changjun Yin

**Affiliations:** State Key Laboratory of Reproductive Medicine, Department of Urology, First Affiliated Hospital of Nanjing Medical University, Nanjing, China; Hormel Institute, University of Minnesota, United States of America

## Abstract

**Objective:**

The aim of this study was to develop a follow-up strategy based on the new model to reduce unnecessary prostate biopsies in patients with prostate specific antigen (PSA) ranging from 4 to 10 ng/ml.

**Methods:**

A total of 436 patients with PSA ranging from 4 to 10 ng/ml who had undergone transrectal ultrasound (TRUS)-guided prostate biopsy were evaluated during the first stage. Age, PSA, free PSA (fPSA), digital rectal examination (DRE) findings, ultrasonic hypoechoic mass, ultrasonic microcalcifications, prostate volume (PV) and PSA density (PSAD) were considered as predictive factors. A multiple logistic regression analysis involving a backward elimination selection procedure was applied to select independent predictors. After a comprehensive analysis of all results, we developed a new model to assess the risk of prostate cancer and an effective follow-up strategy.

**Results:**

Age, PSA, PV, fPSA, rate of abnormal DRE findings and rate of hypoechoic masses detected by TRUS were included in our model. A significantly greater area under the receiver-operating characteristic curve was obtained in our model when compared with using PSA alone (0.782 vs. 0.566). Patients were grouped according to the value of prostate cancer risk (PCaR). In the second stage of our study, patients with PCaR>0.52 were recommended to undergo biopsies immediately while the rest of the patients continued close follow-up observation. Compared with the first stage, the detection rate of PCa in the second stage was significantly increased (33.0% vs 21.1%, p = 0.012). There was no significant difference between the two stages in distribution of the Gleason score (p = 0.808).

**Conclusions:**

We developed a follow-up strategy based on the new model, which reduced unnecessary prostate biopsies without delaying patients’ diagnoses and treatments.

## Introduction

Prostate specific antigen (PSA) is widely used for the screening of prostate cancer. However, an increasing level of PSA can also been seen in benign prostatic hyperplasia (BPH) and prostatitis, which questioned the specificity of PSA in predicting prostate cancer [1]. In patients with PSA levels of 4 to 10 ng/ml, the detection rate of PCa was merely 20% or less thus defining the region as a “gray zone” [2]–[4]. Clearly, there is an urgent need for improving the detection rate and reducing unnecessary prostate biopsies in the “gray zone”.

Recently, models combining PSA levels with other independent risk factors had shown advantages in screening PCa by avoiding unnecessary prostate biopsies [5]. However, the famous models based on European and American populations are not suitable for Chinese males due to population heterogeneity [6], [7]. Moreover, to the best of our knowledge model that was specially designed to increase the PCa detection rate in the PSA “gray zone” was far from satisfactory.

Therefore, we developed a new prostate cancer risk assessment model applicable to patients with PSA levels of 4 to 10 ng/ml. With the help of the new model, we further formulated a reasonable follow-up strategy to increase PCa detection rate and reduce unnecessary prostate biopsies.

## Methods

### Ethics statement

This study was approved by the institutional review board of the First Affiliated Hospital of Nanjing Medical University. Written informed consent was obtained from all patients with regard to the storage of their information for the purpose of research. All research procedures were conducted in accordance with the Declaration of Helsinki.

### First stage

The first stage included 436 patients, who had an elevated PSA level ranging from 4 to 10 ng/ml and had undergone a transrectal ultrasound (TRUS)-guided prostate biopsy at the First Affiliated Hospital of Nanjing Medical University between July and September of 2009. Age, PSA, free PSA (fPSA), digital rectal examination (DRE) findings and other clinical information were recorded in detail. Transrectal ultrasound (TRUS) guided examinations were performed on each patient. The prostate volume (PV) was calculated by TRUS using the formula PV = 0.52× transverse diameter × anteroposterior diameter × cephalocaudal diameter. Additionally, we also recorded hypoechoic lesions and microcalcifications in TURS. PSA density (PSAD) was defined as the ratio of PSA to PV. The free/total PSA ratio (f/t) indicated the percentage of fPSA in total PSA. Prostate biopsies were conducted as 13 cores, including the conventional systemic 12-core biopsy in addition to a special core. The additional core was derived from the hypoechoic lesion under the ultrasound or the apex of the prostate.

To select independent predictors of prostate cancer in the model-building set, the multiple logistic regression analysis with a backward elimination selection procedure was applied. Parameters showing significant differences (p<0.05) were included into a normogram for positive biopsy. An equation for prostate cancer risk (PCaR) was developed based on the final logistic regression model. We appraised the diagnostic efficiency via the Receiver Operating Curve. Based on the value obtained from the PCaR, patients were classified into two risk groups.

### Second stage

In this stage, we prospectively evaluated 188 patients with PSA in the “gray zone” using our model. Patients in the high-risk group received prostate biopsies immediately and the low-risk group was advised to continue careful observation and active 3-month follow-ups. Finally, we compared the detection rate of PCa and distribution of the Gleason Scores between the two stages.

### Statistical analysis

Statistical analysis was performed using SPSS 18.0 software and R version 2.15.0 (http://www.r-project.org/). Differences between the characteristics of patients were analyzed using the student's t-test for continuous variables and the chi-square test for categorical variables. A receiver-operating characteristic curve was used to evaluate the effectiveness of the model and other parameters such as PSA and PSAD. Multiple logistic regression analysis with a backward elimination selection procedure was used to select potential predictors and develop the new model. The Hosmer-Lemeshow test using the concordance index on 100 bootstrapped re-samples was used for validation of the predictive accuracy model. A P value <0.05 was considered statistically significant.

## Results

A total of 436 patients were retrospectively assessed during the first stage, with 21.1% (92/436) positive biopsy results. The demographic characteristics of the study cohort are detailed in **Table 1**. Patients in the PCa group were older (70.2±6.7 vs. 66.3±8.7; p<0.001). Moreover, statistical significant differences were found in fPSA (p = 0.014), PV (p<0.001), PSAD (p<0.001), f/tPSA (p = 0.001), DRE findings (p<0.001) and hypoechoic under transrectal ultrasound findings (p<0.001) between the PCa and non-PCa groups. However, no significant difference was observed in PSA levels (7.3±1.6vs. 7.0±1.6; p = 0.060).

**Table 1:** Characteristics of the patient cohort in the first stage of the study.

variables	Pca	Non-Pca	p
	N(%)	N(%)	
No. of subjects	92(21.1)	344(78.9)	
Age	70.2±6.7	66.3±8.7	<0.001^a^
PSA	7.3±1.6	7.0±1.6	0.060^a^
fPSA	1.0±0.5	1.2±0.8	0.014^a^
PV	33.2±12.7	40.1±17.3	<0.001^a^
PSAD	0.25±0.10	0.20±0.10	<0.001^a^
f/t	0.14±0.06	0.17±0.10	0.001^a^
DRE			<0.001^b^
Normal	68(17.7)	316(82.3)	
Abnormal	24(46.2)	28(53.8)	
Hypoechoic#			<0.001^b^
No	55(16.8)	273(83.2)	
Yes	37(34.3)	71(65.7)	
Microcalcification#			0.511^b^
No	70(21.9)	249(78.1)	
Yes	22(18.8)	95(81.2)	

Values are mean ± SD and number (percent). DRE, digital rectal examination; PSA, prostate-specific antigen; fPSA, free prostate-specific antigen; PSAD, prostate-specific antigen density and PV, prostate volume;

# Hypoechoic areas and microcalcification were detected by ultrasound.

a .Student's t-test for age, PSA, fPSA, PV, PSAD and f/t distributions between Pca and Non-Pca groups.

b . Two-sided χ2-test or Fish's exact test for DRE findings, Hypoechoic, and Microcalcification between Pca and Non-Pca groups.

On multivariate analysis, age, PSA, fPSA, PV, PSAD, f/t, hypoechoic, DRE findings and microcalcification were included in our logistic analysis. After a backward elimination selection procedure, six predictors showed significant differences (PSA, PV, hypoechoic, abnormal DRE, age and fPSA) indicating that they were potential predictors for initial prostate biopsy. **Table 2**.

**Table 2:** Multivariate analysis of the predictors of prostate cancer.

Variables	B	OR	95% CI for OR		p#
			Lower Limit	Upper Limit	
intercept	−5.62				<0.001
PSA#	0.352	1.422	1.186	1.704	<0.001
PV#	−0.043	0.958	0.938	0.979	<0.001
Hypoechoic#	0.822	2.275	1.307	3.961	0.004
Abnormal DRE#	1.377	3.964	1.988	7.903	<0.001
Age#	0.094	1.098	1.058	1.141	<0.001
fPSA#	−1.224	0.294	0.157	0.551	<0.001
PSAD	−0.815	0.443	0.127	0.864	0.926
f/t	−1.598	0.202	0.084	0.773	0.545
Microcalcification	−0.266	0.766	0.416	1.413	0.394

# Age, PSA, fPSA, PV, PSAD, f/t, hypoechoic, DRE findings and microcalcification were included in our logistic analysis with a backward elimination scheme. Six predictors showed significant difference (p<0.05) and were included into an equation for prostate cancer risk (PCaR).

**Figure 1:**
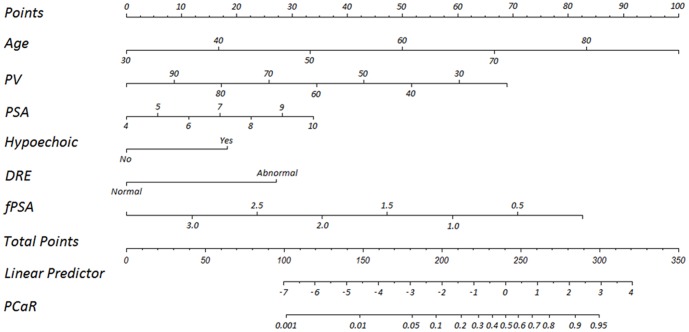
Nomogram for predicting a positive prostate biopsy. Locate patient values on each axis, and compare to the ‘Point’ axis to determine how many points are attributed to each variable. Then, locate the sum of the points for all variables on the ‘Total Points’ line to determine the individual probability of prostate cancer on the ‘PCaR’ line.

**Figure 2:**
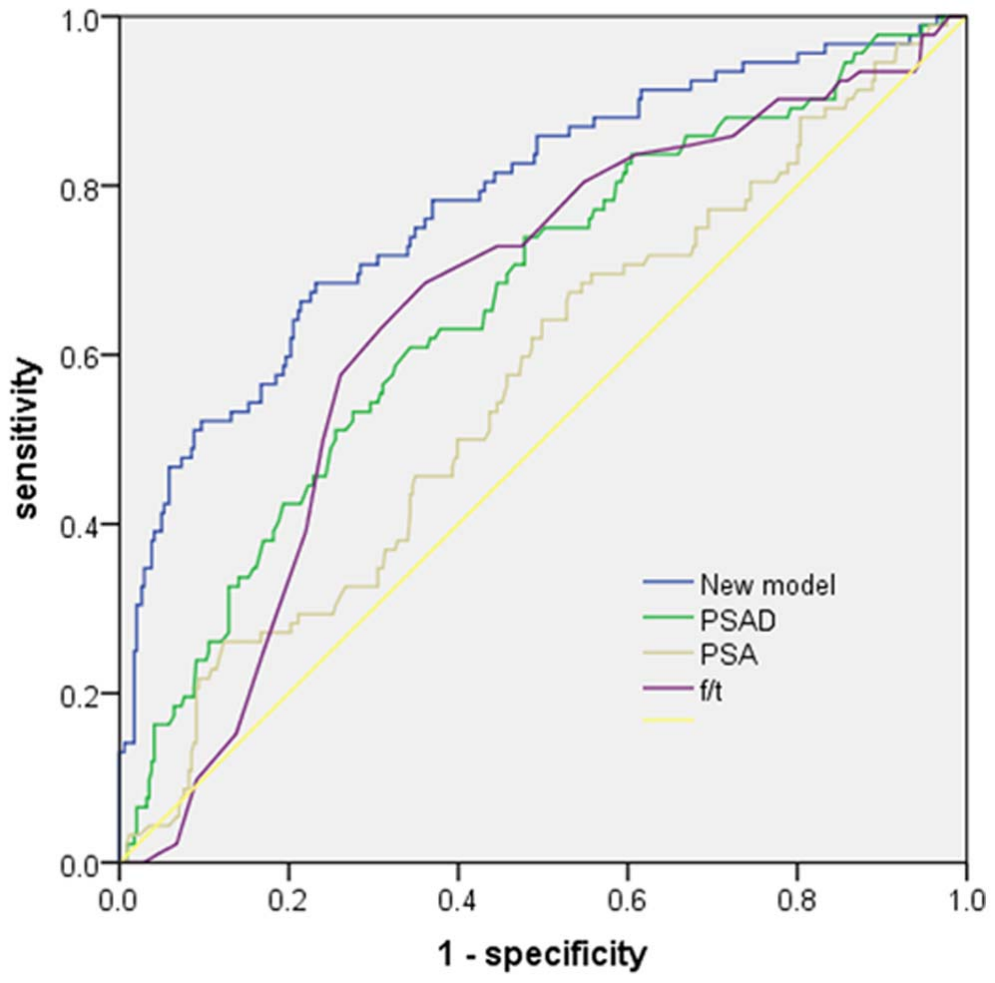
ROC of our new model, PSA, PSAD and f/t. The AUC of these predictors were 0.789, 0.566, 0.664 and 0.654 respectively.

**Figure 3:**
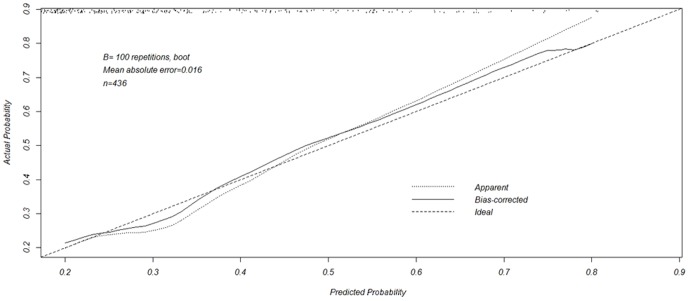
Validation of the predictive accuracy (78.9%).

Thus, the model was created based on the results of the logistic analysis. PSA, PV, hypoechoic, abnormal DRE, age and fPSA were finally included to build the model. The equation for prostate cancer risk (PCaR) was defined as follows:
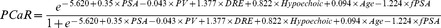



Then, we developed a nomogram resulting from the graphical representation of multivariate regression analysis of the studied variables as shown in **Figure 1**. A remarkable rise of the area under the curve (AUC) of the receiver-operating characteristic curve for the new model (0.789) was observed when compared with conventional clinical parameters such as PSA (0.566), PSAD (0.664) and f/t (0.654) as shown in **Figure 2**. Validation of our new model is shown in **Figure 3**. Using bootstrapping, the predictive accuracy calculated by the Hosmer's concordance index was estimated as 0.789.

Considering that a higher sensitivity should be maintained in detecting PCa, we determined the cutoff value at 0.52, which provided a higher sensitivity of more than 85.0% (87.0% precisely), and the specificity was 45.6%, which was much higher than the specificity of PSA, PSAD and f/t at the same sensitivity level (20.0%, 29.6% and 27.6% respectively). Patients with PCaR>0.52 were entered into the high-risk group, while other the patients were entered into the low-risk group. The detection rate of PCa in the low-risk group was significantly lower than in the high-risk group (6.8% vs 32.0%, p<0.001). Due to the extraordinary low detection rate in the low-risk group, active surveillance every 3 months was recommended.

In the second stage, 188 patients was recruited and evaluated by our model from November 2012 to June 2013. The patients in the high-risk group underwent biopsy. The characteristics of the study cohort in two stages are shown in **Table 3**. The positive rate was improved from 21.1% to 33.0% (p = 0.012) compared to the retrospective cohort. No significant difference in basic conditions and distribution of Gleason scores was found between the two groups (p = 0.808).

**Table 3:** Comparison of patients' characteristics between two stages.

variables	Before new model		Using new model*		p
	N(%)	Median	N(%)	Median	
No. of subjects	436		188		
Age	67.1±8.4	69	67.8±8.3	68	0.380^a^
PSA	7.0±1.6	7.05	7.1±1.7	7.18	0.584^a^
fPSA	1.2±0.8	1.05	1.2±0.6	1.13	0.887^a^
PV	39.0±16.7	35.6	40.0±17.5	37.19	0.507^a^
PSAD	0.2±0.1	0.1955	0.2±0.1	0.1996	0.881^a^
f/t	0.2±0.1	0.15	0.2±0.1	0.16	0.951^a^
PCaR	0.6±0.2	0.61	0.6±0.3	0.63	0.601^a^
Abnormal DRE	52(11.9)		23(12.2)		0.894^b^
Hypoechoic	108(24.8)		58(30.9)		0.116^b^
No. of subjects with biopsies	436(100)		112(59.6)		<0.001^b^
Positive cases	92(21.1)		37(33.0)#		0.012^b^
Gleason score					0.808^b^
6	62(67.4)		21(62.2)		
7	20(21.7)		10(27.0)		
≧8	10(10.9)		6(10.8)		

*This table showed most patients' basic information at their first visits (patients with watchful waiting) or latest visits before biopsies (patients with biopsies).

# In stage two, positive rate was discussed in patients with biopsies only.

a . Student's t-test for age, PSA, fPSA, PV, PSAD, f/t and PCaR distributions between two stages.

b . Two-sided χ2-test or Fish's exact test for DRE findings, Hypoechoic, No. of subjects with biopsies, Positive cases and Gleason score between two stages.

Along with the follow-up of patients in the low-risk group, the value of PCaR in 5 patients surpassed 0.52, which is an indication for biopsy. After obtaining informed consent, all of them underwent biopsy immediately, and 3 of the patients had positive biopsy results.

## Discussion

With PSA routine screening, the number of patients diagnosed with PSA in the “gray zone” has been increasing rapidly. However, due to conditions such as cancer, inflammation and benign hyperplasia with overlapping low PSA levels, the positive rate hovered around approximately 20% [8]. To improve the detection rate of prostate cancer, several upgraded predictors such as PSAD, %fPSA, PSAV and PSATZ were introduced [9]–[11]. However, most of these univariate parameters have evolved from PSA, therefore making them provincial.

Previous studies have demonstrated that predictive models, covering clinical, laboratory and other parameters, such as the prostate cancer prevention trial (PCPT) and the European randomized study of screening for prostate cancer (ERSPC) can improve the accuracy of prostate cancer detection to various degrees [12]. Unfortunately, the incidence of prostate cancer among various regions differed, which imposed restrictions on applying the methods in Chinese men [13]. Furthermore, due to the particularities of patients with PSA levels of 4 to 10 ng/ml, multivariate analysis in patients with PSA levels in the “gray zone” rather than in all patients with elevated PSA levels may be more accurate. Thus, it is obvious that a model particularly designed for patients in the “gray zone” can gain further insights. As far as we know, this is the first model designed specifically for patients with PSA levels from 4 to 10 ng/ml in a Chinese population to improve the accuracy of prostate cancer detection.

Interestingly, we noticed that total PSA exhibited no significant difference on univariate analysis between the cancer and non-cancer groups. One reasonable explanation may be that most of the patients with PSA levels in the “gray zone” actually had inflammation, benign hyperplasia or a low risk for cancer, thereby diminishing the difference [14]. Considering these results, we insist that using PSA alone was far from satisfactory. Therefore, we combined other factors to set up our new model.

**Figure 4:**
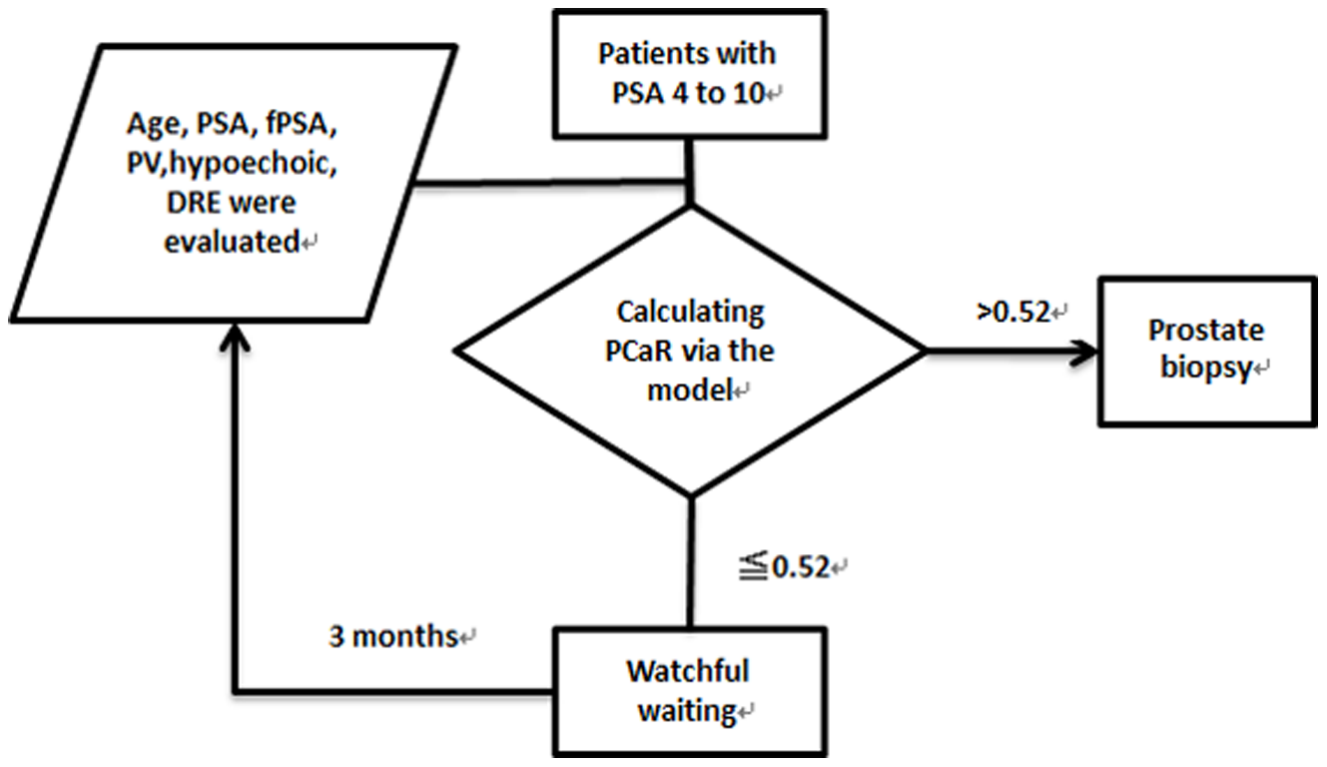
Management for patients with PSA from 4 to 10 ng/ml based on our new model.

Compared with PSA alone, our newly developed model enlarged AUC from 0.566 to 0.789, which means the accuracy for predicting PCa risk was substantially improved. Meanwhile, it was also more efficient than commonly used upgraded predictors such as PSAD and f/tPSA. According to the PCaR values, patients with PCaR>0.52 were placed into the high-risk group while the remaining patients were entered into the low-risk group. A notably higher percentage of prostate cancer was revealed in 247 patients in the high-risk group than in the 189 patients in the low-risk group (32.0% vs 6.8%, p<0.001). Due to the high proportion (93.2%, 176/189) of irrelevant neoplasms in the low-risk group, conducting biopsies in this group was unwise. By applying our new model, a follow-up strategy for patients with PSA levels in the “gray zone” was developed. Patients in the high-risk group were supposed to receive prostate biopsies immediately while patients in the low-risk group were recommended to be reassessed 3 months later, as shown in **Figure 4**. Theoretically, in the first stage 189 (43.3%) patients in the low-risk group, including 6.8% patients with prostate cancer, were supposed to receive active surveillance.

In the second stage of our study, we verified our follow-up strategy prospectively in 188 patients with PSA levels in the “gray zone”. Only 112 (59.6%) patients underwent prostate biopsies due to their higher PCaR values. Meanwhile, the positive rate of PCa was improved significantly (33.0% vs 21.1%, p = 0.012). Compared to the first stage, the distribution of the Gleason score (GS) remained almost the same (p = 0.808), indicating that even patients with suspicious conditions can be detected, which further proved the sensitivity and specificity of our model. We also discovered a higher percentage of patients with median and high GS (7 or more). In another words, a certain number of patients with low-risk prostate cancer (GS = 6) could benefit from our model by avoiding further aggressive management such as biopsies or radical prostatectomies because they could enjoy favorable life expectancies without any treatment [15]–[17] considering the average age and life expectancy of them [18], [19].

The PCaR values of 5 patients increased and surpassed the threshold at their return visits. We performed biopsies immediately and 3 out of the 5 (60%) patients were diagnosed with PCa. However, whether the rise in PCaR value indicates the risk of prostate cancer occurrence needs further investigation.

The present study has several limitations. First, the total number of patients recruited was 624, which is relatively smaller than PCPT and ERSPC. However, to the best of our knowledge, this study included the largest number of patients with PSA levels in the “gray zone” in a Chinese population. Second, other models that predict the risk of prostate cancer among men with PSA levels between 4 and 10 ng/ml exist [20], [21]. Nevertheless, compared with other models, our model had a higher AUC (0.789 vs. 0.73 and 0.759 respectively) and higher predictive accuracy based on PSA alone (0.223 vs. 0.11 and 0.182 respectively), which indicated that our model is more reliable and could identify more patients with a high risk for prostate cancer as shown in **Table 4**. Third, some novel biomarkers such as p2PSA and PCA3 [22]were not included in the new model due to the restriction of healthcare in China. It is rather difficult to obtain these new biomarkers during physical examination routinely in most areas.

**Table 4:** Comparison between our model and other earlier models.

Study	n	Positive rate(%)	AUC for Model	AUC for PSA	Increase in AUC vs. PSA alone
Our model	436	21.1	0.789	0.566	0.223
Mark Garzotto et al	1237	25.0	0.73	0.62	0.11
Jae Hyun Ahn et al	1171	21.8	0.759	0.577	0.182

## Conclusions

In conclusion, we developed the first model to predict the positive risk in patients with PSA levels between 4 and 10 ng/ml in a Chinese population. Along with that, we developed a follow-up strategy, which reduced unnecessary prostate biopsies and increased the detection rate of PCa without delaying patient diagnosis and treatment.

## Supporting Information

File S1 All data used in this article was contained in this file.
(XLSX)
